# Clinical Improvements in Comorbid Gambling/Cocaine Use Disorder (GD/CUD) Patients Undergoing Repetitive Transcranial Magnetic Stimulation (rTMS)

**DOI:** 10.3390/jcm8060768

**Published:** 2019-05-30

**Authors:** Stefano Cardullo, Luis Javier Gomez Perez, Linda Marconi, Alberto Terraneo, Luigi Gallimberti, Antonello Bonci, Graziella Madeo

**Affiliations:** 1 Human Science and Brain Research, Novella Fronda Foundation, Piazza Castello, 35141 Padua, Italy; stefano.cardullo@gmail.com (S.C.); luigomper@gmail.com (L.J.G.P.); linda.marconi22@gmail.com (L.M.); alberto.terraneo@gmail.com (A.T.); luigi.gallimberti@studiogallimberti.it (L.G.); antonello.bonci@nih.gov (A.B.); 2 Intramural Research Program, National Institute on Drug Abuse, National Institutes of Health, Baltimore, MD 21224, USA; 3 Solomon H. Snyder Department of Neuroscience, Johns Hopkins University School of Medicine, Baltimore, MD 21205, USA; 4 Department of Psychiatry and Behavioral Sciences, Johns Hopkins University School of Medicine, Baltimore, MD 21287, USA

**Keywords:** gambling disorder (GD), cocaine use disorder (CUD), craving, repetitive transcranial magnetic stimulation (rTMS), Gambling-Symptoms Assessment Scale (G-SAS), dorsolateral prefrontal cortex (DLPFC)

## Abstract

(1) Background: Pathological gambling behaviors may coexist with cocaine use disorder (CUD), underlying common pathogenic mechanisms. Repetitive transcranial magnetic stimulation (rTMS) has shown promise as a therapeutic intervention for CUD. In this case series, we evaluated the clinical effects of rTMS protocol stimulating the left dorsolateral prefrontal cortex (DLPFC) on the pattern of gambling and cocaine use. (2) Methods: Gambling severity, craving for cocaine, sleep, and other negative affect symptoms were recorded in seven patients with a diagnosis of gambling disorder (South Oaks Gambling Screen (SOGS) >5), in comorbidity with CUD, using the following scales: Gambling-Symptom Assessment Scale (G-SAS), Cocaine Craving Questionnaire (CCQ), Beck Depression Inventory-II (BDI-II), Self-rating Anxiety Scale (SAS), and Symptoms checklist-90 (SCL-90). The measures were assessed before the rTMS treatment and after 5, 30, and 60 days of treatment. Patterns of gambling and cocaine use were assessed by self-report and regular urine screens. (3) Results: Gambling severity at baseline ranged from mild to severe (mean ± Standard Error of the Mean (SEM), G-SAS score baseline: 24.42 ± 2.79). G-SAS scores significantly improved after treatment (G-SAS score Day 60: 2.66 ± 1.08). Compared to baseline, consistent improvements were significantly seen in craving for cocaine and in negative-affect symptoms. (4) Conclusions: The present findings provide unprecedent insights into the potential role of rTMS as a therapeutic intervention for reducing both gambling and cocaine use in patients with a dual diagnosis.

## 1. Introduction

Pathological gambling behaviors frequently co-occur with substance use [1]. A prevalence rate of 10.3% has been reported for gambling disorder (GD) diagnosis in substance use disorders (SUD) patients [2,3], while GD patients have a 57.5% prevalence for substance-related disorder comorbidity [4,5]. Despite the differences observed in cue and stress-related craving, GD shares some of the neurobiological substrates, psychological processes, and behavioral manifestations of SUDs [6,7,8]. Compelling evidence supports that both GD and SUDs are sustained by impaired neuroplasticity and dysfunctions within reward, stress and cognitive-control systems. These abnormalities underline the core clinical manifestations [9], such as compulsive gambling or compulsive drug consumption, craving, altered reward sensitivity, and impaired self-control and decision-making processes [10]. Moreover, diminished executive functions (e.g., diminished response inhibition, cognitive flexibility) in GD patients compared to healthy controls seem related to differential functioning of the cognitive control circuit involving the dorsolateral prefrontal cortex (DLPFC) and the anterior cingulate cortex (ACC) [11].

Neuromodulation approaches could represent a promising therapeutic intervention for GD [12], able to restore the abnormalities in cognitive motivational-behavioral and executive functions. Non-invasive brain stimulation techniques, like transcranial magnetic stimulation (TMS), may enhance cognitive control through DLPFC stimulation, thus restoring some of the core symptoms of either GD and SUDs [13,14,15,16]. Specifically, repetitive TMS (rTMS) stimulating the left DLPFC has been shown to be effective in reducing craving in substance-related disorders [17,18,19,20] and to improve cognitive functioning [21]. Currently, a limited number of TMS pilot studies in GD has investigated whether TMS may be a promising treatment for GD, often providing conflicting results. For instance, in a sham-controlled cross-over high-frequency rTMS study targeting the left DLPFC, active rTMS diminished cue-induced craving compared to sham rTMS [22]. Conversely, low-frequency rTMS (1 Hz) over the right DLPFC had similar effects as sham stimulation on craving, even though with a large placebo effect [23]. In another trial involving nine gamblers, a single session of high frequency rTMS reduced desire to gamble, whereas continuous theta burst stimulation (cTBS) reduced blood pressure, with no effects on craving [24]. In this same study no effects on impulsive behavior (delay discounting) and Stroop interference were evident. Yet, the only deep transcranial stimulation pilot study at 1 Hz targeting prefrontal regions had no effects on pathological gambling [25]. This discrepancy might be related to small sample size, variability of study design, stimulation parameters, and protocols [8]. Moreover, most of them investigated the effects of a single TMS session, which is unlikely to influence a complex behavioral pattern, such as gambling. Nevertheless, from these few studies, it appears that TMS has the ability to alter at least some of the working mechanisms underlying pathological gambling. The abundant evidence on neurobiological and behavioral similarities between GD and SUDs [26] and the potential clinical benefit from cumulative rTMS sessions [27] led us to investigate whether gambling symptoms in patients with cocaine use disorder (CUD) may improve following a high frequency rTMS treatment targeting the left DLPFC.

Here, we report a case series of seven treatment-seeking patients with cocaine use disorder (CUD) comorbid with GD who underwent an rTMS treatment stimulating the left DLPFC. We investigated the clinical changes regarding the patterns of cocaine use, gambling, and accompanying withdrawal symptoms during the treatment.

## 2. Materials and Methods

The study was conducted in accordance with the Declaration of Helsinki and the protocol approved by the Ethics Committee for Psychological Research, University of Padua (Protocol Number: 2551; registration Number: NCT03733821) [28].

### 2.1. Participants and Treatment

Seven male participants diagnosed as suffering from cocaine use disorder (CUD) were selected based on comorbidity with GD, according to DSM-5 [29], from 87 patients recruited for a retrospective observational study investigating sleep disturbances. All participants, treatment seekers for CUD, provided informed consent and underwent an rTMS protocol at a clinic center for addiction treatment in Padua, Italy. During the screening, patients were assessed for substances of abuse pattern and gambling experiences. Only CUD patients comorbid with GD were selected. GD was determined by a clinical interview with an SUD expert psychiatrist combined with a score equal to or greater than 5 on the South Oaks Gambling Screen (SOGS) [30]. All patients were slot machine players, except one, who mainly played online poker. To exclude contraindications to receive rTMS, a TMS safety screening was administered to all patients in line with international recommendations for TMS safety and ethics [31]. Patients with a lifetime history of other psychiatric diseases, including major depression, schizophrenia, bipolar disorder or other psychoses, current alcohol and other substance use disorders (excluding cocaine, tobacco, and caffeine), personality disorders, and unstable medical illness were also excluded from the initial sample. During treatment, cocaine use was monitored by urine drug tests and relapses were reported. The urine drug testing also screened for morphine, methadone, tetrahydrocannabinol (THC), phencyclidine, amphetamine, and methamphetamine. Pharmacological therapy remained unchanged or was not prescribed during the treatment. rTMS treatment was administered to each patient by a trained clinical physiologist using a medical device (MagPro R30) targeting the left DLPFC. Treatment consisted of twice-daily rTMS sessions for the first five consecutive days of treatment, followed by twice-daily rTMS sessions once a week over eight weeks. Protocol parameters, such as stimulation parameters and motor threshold detection, were defined according to the procedures described by Terraneo et al. [18]. To best identify the DLPFC (Montreal Neurological Institute (MNI) coordinates x: −50, y: 30, z: 36), we used an optical TMS navigator (Localite, St. Augustin, Germany) and a magnetic resonance image (MRI) template. The stimulation parameters were: frequency: 15 Hz; intensity: 100% of the motor threshold; 60 impulses per stimulation train; inter-train interval: 15 s; and 40 total trains for a session duration of 13 min. At each session, adverse events, including seizures, syncopes, neurological complications, or subjective complaints about memory, concentration, pain, headache, vertigo, or fatigue were assessed.

Gambling symptomatology was evaluated by Gambling Symptom Assessment Scale (G-SAS) [32]. The G-SAS is a 12-item self-rated scale designed to assess gambling symptom severity and change during treatment with a score ranging from 0 to 4 [32].

Participants were also assessed for craving, subjective sleep quality, depression, anxiety, and other negative affect symptoms. The Cocaine Craving Questionnaire (CCQ) [33] is a 5-item self-report questionnaire measuring five aspects of craving: current intensity, intensity during the previous 24 h, frequency, responsiveness to drug-related conditioned stimuli, and imagined likelihood of use if in a setting with access to drugs [34]. The 19-item Pittsburgh Sleep Quality Index (PSQI) [35] is the most commonly used retrospective self-report questionnaire measuring the self-perceived quality of sleep [36]. It investigates sleep quality, sleep latency, sleep duration, habitual sleep efficiency, sleep disturbances, use of sleeping medications, and daytime dysfunction [37]. The Beck Depression Inventory-II (BDI-II) [38] is a 21-item self-report questionnaire format with four options under each item, ranging from not present (0) to severe (3), measuring depressive symptoms. The Self-rating Anxiety Scale (SAS) [39], is a 20-item measure developed to assess the frequency of anxiety symptoms based on diagnostic conceptualizations. It consists primarily of somatic symptoms. The Symptoms checklist 90-Revised [40] is a 90-item self-report inventory which assesses psychological distress in terms of nine primary symptom dimensions and three summary scores termed global scores. The Global Severity Index (GSI), the outcome of which we used in this study, is the single best indicator of the current level or depth of an individual’s disorder. It combines information concerning the number of symptoms reported with the intensity of perceived distress [41].

The assessments were made at baseline, immediately after completion of the first week of treatment, as well as 30 and 60 days after the beginning of treatment (day 5, day 30, day 60). BDI-II was not included in the assessment on day 5 since it considers changes in the preceding two weeks. Some participants did not complete every scale at every time point, for the main following reasons: clinical response, missing follow-up visit, missing TMS session, and refusal. We included participants who completed outcome measures for at least three time points, including the baseline.

### 2.2. Statistical Analyses

We used repeated-measures analyses of variance (ANOVA) for testing the effect of treatment over time, followed by the Tukey test for post-hoc comparison analysis between timepoints (day 5, day 30, and day 60). Data were expressed as mean ± SD, unless otherwise specified; *α* was set at <0.05, two-tailed. All the analyses were performed using RStudio version 1.1.453 [42] with R version 3.5.0 [43] and the package emmeans [44]. G*Power (ver 3.1.9.4) was used to perform a power analysis and estimate the sample size. For the computation of this estimate we considered the effects we observed in a previous pilot study [18]. Considering an Effect size f(U) = 1.1, an *α* = 0.05, a power (1 − β) = 0.95, one group and four measurements, a sample size of seven patients was required.

## 3. Results

The full demographic and clinical characteristics of the participants are presented in Table 1. The total sample consisted of seven male patients, aged between 30 and 49 (42.14 ± 5.74). Treatment variables are specified in Table 1.

**Figure 1:**
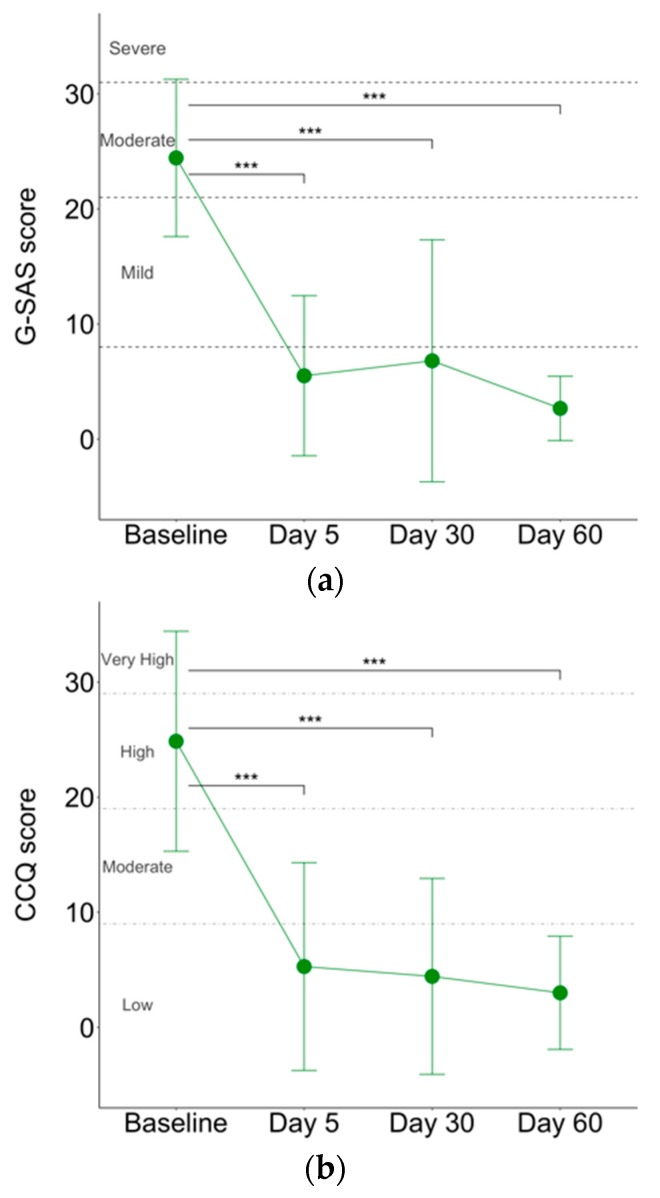
Plot of the means and 95% confidence intervals of (**a**) Gambling-Symptom Assessment Scale (G-SAS) and (**b**) Cocaine Craving Scale (CCQ). *** *p* value < 0.001.

G-SAS scores significantly improved at each time point after the first week of treatment (F(3,22) = 16.71, *p* < 0.001, R^2^ = 0.69; Figure 1a). Pairwise comparisons showed that G-SAS scores at day 5 were significantly lower than those at baseline (t(22) = 5.61, *p* < 0.001). This improvement was maintained at the subsequent timepoints: day 30 (t(22) = 5.07, *p* < 0.001) and day 60 (t(22) = 6.22, *p*< 0.001). Similarly, craving for cocaine, reflected in CCQ scores, significantly decreased over time (F(3,24) = 9.52, *p* < 0.001, R^2^ = 0.54; Figure 1b). Pairwise comparisons showed a significant improvement from baseline to day 5 (t(24) = 4.12, *p* < 0.001) and levels were maintained at day 30 (t(24) = 4.30, *p* < 0.001) and at day 60 (t(24) = 4.60, *p* < 0.001). No significant differences were found when comparing G-SAS and CCQ scores among day 5, day 30, and day 60. Of seven patients, four did not report any relapse in cocaine and in gambling behaviors during the 60 days of treatment.

Similarly, sleep disturbance, depression, anxiety, and the other negative affect symptoms significantly decreased over time as reflected in PSQI (F(3,24) = 5.40, *p* < 0.01, R^2^ = 0.40), BDI-II (F(2,18) = 21.53, *p* < 0.001, R^2^ = 0.70), SAS (F(3,23) = 14.19, *p* < 0.001, R^2^ = 0.64), and GSI (F(3,24) = 7.04, *p* < 0.01, R^2^ = 0.46). The secondary outcome improvement was significant at day 5 of the treatment in comparison to the baseline (PSQI: t(24) = 3.40, *p* = 0.01; SAS: t(25) = 5.68, *p* < 0.001; GSI: t(24) = 3.69, *p* < 0.01) and stable overtime: day 30 (PSQI: t(24) = 3.50, *p* < 0.01; BDI-II: t(18) = 5.40, *p* < 0.001; SAS: t(23) = 4.25, *p* < 0.01; GSI: t(24) = 3.38, *p* = 0.01) and day 60 (PSQI: t(24) = 2.72, *p* = 0.05; BDI-II: t(18) = 5.92, *p* < 0.001; SAS: t(23) = 5.47, *p* < 0.001; GSI: t(24) = 4.05, *p* < 0.01).

### 3.1. Cocaine Use

Five out of seven patients did not use cocaine for the entire duration of the study. One patient used cocaine after the first 15 days of treatment. Another patient used cocaine during the first week and another three times within 60 days of treatment. None of the patients used other drugs included in the urine drug screen.

### 3.2. Safety

No adverse events were reported.

## 4. Discussion

To our knowledge, this is the first study reporting clinical improvements in patients with a dual diagnosis of CUD and GD following an rTMS treatment stimulating the left DLPFC.

We evaluated changes in cocaine use and gambling patterns as well as the accompanying symptoms, including sleep disturbances, mood, anxiety, and other negative symptoms [45,46] in seven patients seeking treatment for CUD.

The severity of the gambling symptoms and cocaine craving ranged from moderate to severe at baseline, as assessed by G-SAS and CCQ. G-SAS and CCQ scores dramatically dropped at the end of the first week of treatment and were stable during the following 60 days of treatment. Moreover, four out of seven patients did not show either cocaine use or relapse of gambling behaviors; one patient reported four gambling episodes during the first month of treatment (total 454 €), without any cocaine use; one patient reported a single gambling episode during the first week of treatment (150 €) associated with cocaine use, followed by another three uses within 60 days of treatment; one patient reported several cocaine uses after the first 15 days of treatment but no gambling behavior. The improvement of cocaine-related symptoms is in line with previous findings showing that high-frequency left-DLPFC-rTMS stimulation is effective in diminishing cocaine craving [17,19,47] and intake [18]. Neuroimaging studies have shown common activation abnormalities in regions of the mesolimbic reward system in patients with substance-related disorders and GD [48,49,50,51,52], supporting the concept of a common etiology for both disorders. The repeated exposure to addictive stimuli induces plastic changes within reward pathways leading to the hypersensitivity of the brain reward system to the addictive stimulus itself and addiction-related cues. This process is instrumental for conditioning to occur, since previous neutral stimuli can acquire an incentive salience and promote habit formation with repeated exposure to the cues [53,54]. This shift from goal-directed behavior to more habitual responding specifically involves the dorsal striatum (DS) [54], a region implicated in reward, habit learning, and compulsive behaviors. The shift from prefrontal cortical to striatal control may emphasize maladaptive neuroplasticity changes in both GD and SUDs [8]. Accordingly, we observed clinical improvement of either gambling or cocaine-related symptoms. So far, only a few pilot studies have explored the effects of TMS on gambling urges and behavior [22,23,24]. One of these studies evaluated the effect of deep TMS over the DLPFC for 15 days reporting an improvement of craving scores as opposed to a retention of gambling behavior in all patients [25]. Conflicting results might reflect differences in stimulation parameters and treatment conditions. Of note, the clinical improvement in our small cohort of patients was maintained for the whole 60 days period of treatment. Moreover, we observed a significant change of accompanying symptoms to addictive behaviors, including sleep disturbances, depression, anxiety, and negative-affect symptoms, as demonstrated by the improvement of PSQI, BDI-II, SAS, and SCL-90-R scores during the treatment period. Several findings already support a beneficial effect of rTMS for primary sleep disorders and for sleep disturbances comorbid with other neuropsychiatric disorders [55,56,57,58]. To our knowledge, this is one of the first studies reporting a beneficial effect on accompanying symptoms to addictive behaviors and, thus, the results have to be interpreted cautiously. We can argue that anxiety, depressive, and negative-affect symptoms may indirectly benefit from the improvement of the patterns of use and gambling, including the diminished craving and possibly a better executive function control. In our setting with a small sample size, no control group, or a sham-controlled double blind design, we cannot rule out a possible placebo effect, as previously reported in another study using low frequency rTMS targeting the right DLPFC [23]. However, the longer period of observation and the cumulative rTMS sessions may support a beneficial effect related to the neuromodulatory intervention. Certainly, rigorously conducted clinical trials with sham-controlled double-blind designs are needed to investigate whether TMS protocols have the potential to be effective to treat cognitive dysfunctions, reduce craving, and/or the gambling behavior in patients with GD.

## Figures and Tables

**Table 1:** Demographic and clinical characteristics of the participants.

Variables	*n* = 7 ^1^
Age (years)	42.14 (5.74)
Education (years)	12 (3.19)
Cocaine: Age at first experience (years)	27.71 (9.06)
Cocaine: Age at addiction (years)	37.42 (7.51)
Gambling: Age at first experience (years)	19.14 (5.61)
Gambling: Age at addiction (years)	27 (8.31)
SOGS score	9.14 (1.88)
G-SAS score at baseline	24.42 (6.84)
CCQ score at baseline	24.85 (9.56)
PSQI score at baseline	10 (3.25)
BDI-II score at baseline	22.42 (6.16)
SAS score at baseline	50.71 (4.76)
GSI score at baseline	68.32 (10.2)

^1^ Data are presented as mean (standard deviation). SOGS: South Oaks Gambling Screen; G-SAS: Gambling-Symptom Assessment Scale; CCQ: Cocaine Craving Questionnaire; PSQI: Pittsburgh Sleep Quality Index; BDI-II: Beck Depression Inventory-II; SAS: Self-rating Anxiety Scale; GSI: Global Severity Index of the Symptoms checklist 90—Revised.
